# Double Distal Perfusion Catheters for Severe Limb Ischemia on the IABP Side in Patients Who Received Femoro-Femoral VA-ECMO With IABP

**DOI:** 10.3389/fmed.2021.692399

**Published:** 2021-08-25

**Authors:** Meng Xin, Liangshan Wang, Xiaqiu Tian, Dengbang Hou, Hong Wang, Jiangang Wang, Ming Jia, Xiaotong Hou

**Affiliations:** Center for Cardiac Intensive Care, Beijing Anzhen Hospital, Capital Medical University, Beijing, China

**Keywords:** distal perfusion catheter, extracorporeal membrane oxygenation, intra-aortic balloon pump, osteofascial compartment syndrome, severe limb ischemia

## Abstract

**Background:** Limited research is available on the pattern of double distal perfusion catheters in patients on venoarterial extracorporeal membrane oxygenation (VA-ECMO) with an intra-aortic balloon pump(IABP). Here, we compared the outcomes of a double distal perfusion catheter and conventional treatment in patients who received VA-ECMO with IABP and had severe lower limb ischemia on the IABP side.

**Methods:** We reviewed the data of 15 adult patients with postcardiotomy cardiogenic shock who received VA-ECMO via femoral cannulation combined with an IABP in the contralateral artery that was complicated with severe acute limb ischemia (ALI) on the same side as the IABP between January 2004 and December 2016. Patients underwent symptomatic treatment (conventional group, *n* = 9) and double distal perfusion catheterization treatment (DDPC group, *n* = 6). ALI was monitored using near-infrared spectroscopy placed on both calves after double distal perfusion catheters. The outcomes were compared.

**Results:** All 6 patients who underwent double distal perfusion catheters were successfully decannulated without the development of osteofascial compartment syndrome, amputation, or bleeding and infection of the double distal perfusion catheters. The number of patients who weaned from extracorporeal membrane oxygenation successfully in the DDPC and conventional groups was 6 (100%) and 3 (33%, *p* = 0.028), respectively. The in-hospital mortality rates were 17% and 89% for the DDPC and conventional groups, respectively (*p* = 0.011).

**Conclusions:** DDPC can be considered a strategy for severe limb ischemia on the IABP side in patients who received femoro-femoral VA-ECMO with IABP.

## Introduction

Extracorporeal membrane oxygenation (ECMO) via femoral arteriovenous cannulation is an established option for adult patients with refractory cardiogenic shock after cardiac surgery. However, acute limb ischemia (ALI) is a common complication and sometimes requires fasciotomy or amputation and is a significant predictor of mortality (1, 2). Although it is controversial whether patients with intra-aortic balloon pumps (IABPs) combined with ECMO have better prognoses (3, 4), they might have an increased risk of ALI (5). ALI is a complication on both the ipsilateral side of the arterial cannula (ECMO side) as well as the contralateral side (IABP side). Conservative treatment is often ineffective in patients with severe ALI, and IABP may even need to be removed. Insertion of a distal catheter may be an effective means to improve the blood supply of the ischemic lower extremity and reserve IABP.

**Figure 1:**
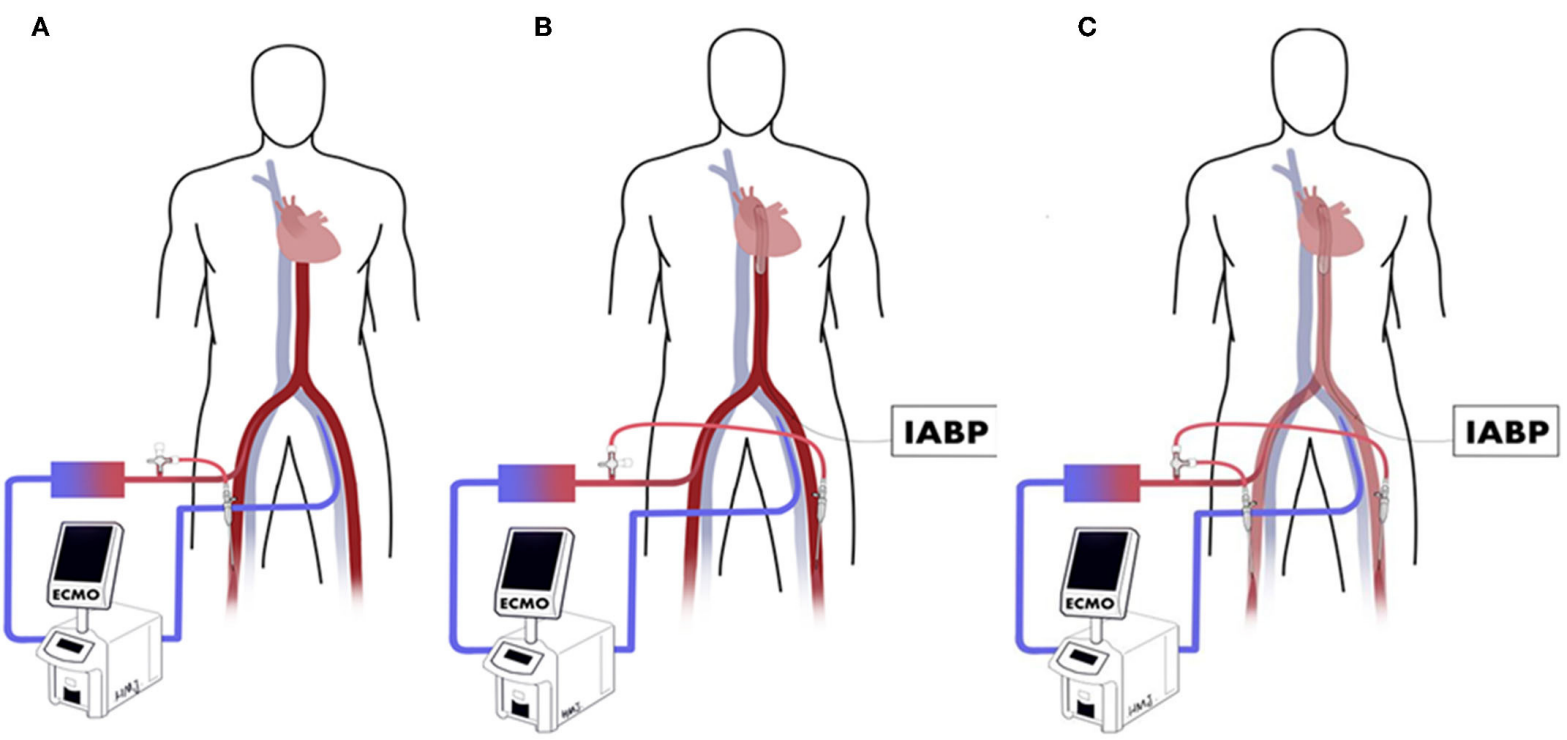
**(A)** ECMO side distal perfusion catheter (EDPC). **(B)** Perfusion catheter inserted to the distal of IABP side called after IABP side distal perfusion catheter (IDPC). **(C)** Double distal perfusion catheter (DDPC).

We describe 3 types of establishing perfusion catheters to relieve distal ischemia (Figure 1). The oxygenated blood is diverted from the arterial cannula to the distal limb of the ECMO side using a perfusion catheter, called the ECMO side distal perfusion catheter (EDPC). Some centers insert EDPC as a preventive routine to decrease limb ischemia (2, 6), while others implant it as treatment following ALI (7, 8). When patients with EDPC develop IABP side limb ischemia, another distal perfusion catheter is inserted distally on the IABP side, called a double distal perfusion catheter (DDPC). In the present study, we compared the outcomes of DDPC and conventional treatment in ECMO patients with ALI on the IABP side.

## Methods

### Study Population

Between January 2004 and December 2016, 451 patients required VA-ECMO support following postcardiotomy cardiogenic shock (PCS), of which 245 patients required combined treatment with IABP. Fifteen patients (6%) who were diagnosed with severe ALI on the IABP side were retrospectively enrolled in this study. Before January 2015, 9 patients underwent conventional treatment. Since January 2015, 6 patients underwent treatment with DDPC. Severe ALI was classified according to the Rutherford system as IIA, IIB or III (9). Category I was excluded because of better prognosis. The study was approved by the institutional ethics committee/review board of the Beijing Anzhen Hospital, Capital Medical University, and the requirement for informed patient consent was waived in view of the retrospective nature of the study.

### ECMO Implantation Techniques

The VA-ECMO was placed by trained ECMO team members. ECMO cannulae (Biomedicus, Medtronic; Minneapolis, MN, USA) were inserted through the femoral artery and femoral vein. A 6-Fr EDPC was inserted at the time of ECMO initiation to preserve limb perfusion. An IABP catheter (Datascope Corp., Fairfeld, NJ, USA) was placed percutaneously through the contralateral femoral artery.

### Patient Management

Detailed management strategies for patients have been previously described (10). ECMO blood flow was adjusted to maintain a mixed venous oxygen saturation (SvO2) level of 70%. Blood circulation of the lower limbs was observed continuously by trained ICU staff during ECMO support. Medial or/and lateral incisions of a minimum of 15 cm were made when acute compartment syndrome developed (intracompartmental pressure ICP >25 mmHg). Amputation was considered when ischemic tissue was subjected to unmanageable infections and when ischemic rest pain or tissue loss could not be restored by any surgical or non-surgical approaches.

A heparin bolus (5,000 IU) was injected before ECMO insertion. After surgical bleeding was controlled, unfractionated heparin was infused continuously as early as possible to maintain an activated clotting time of 160–180 s. When SLI occurred on the IABP side, intravenous prostaglandin therapy or sympathicolysis was initiated. The vasoconstrictors were reduced gradually following hemodynamic stability. Upon failure to relieve ischemia, IABP was removed (conventional group). After January 2015, we began to insert DDPC in such patients (DDPC group).

### DDPC Insertion

A 2–3-cm incision was made 1 cm below the midpoint of the groin. The vasculo-neural sheath was dissected after the subcutaneous tissues and muscles were released. A 6-Fr distal perfusion catheter (Transradial Kit, Cordis Corporation, Miami Lakes, FL, USA) was inserted into the superficial femoral artery at the end of the IABP artery. The distal perfusion catheter was connected to the side hole of the arterial cannula of the ECMO circuit. DDPC decannulation was performed while ECMO was weaning.

### NIRS

Continuous monitoring of limb perfusion began immediately after ALI was diagnosed and measured using bilateral near-infrared spectroscopy (NIRS). The Oximeter sensor pads were placed on the bilateral lower limbs midway between the knee and ankle.

### Data Collection

All clinical variables of patients were recorded in our institutional database. Tissue saturation (StO2) was detected using near-infrared spectroscopy in the ICU, and persistent ALI was diagnosed until stable conditions were achieved. In addition to lactate and muscle injury markers, StO2 was recorded when ALI was diagnosed 6 and 24 h later. Patients also underwent measurement of the ankle brachial index (ABI) of both legs after DDPC was placed and during the follow-up measurements until ECMO and IABP were removed. Clinical indications of hypoperfusion were also recorded, including cold limbs, mottled skin, and pulseless Doppler signaling after DDPC.

### Statistical Analysis

SPSS software (IBM Corp., SPSS Version 25, Armonk, NY, USA) was used for statistical analysis. Baseline classification data were expressed as percentages, and continuous data were expressed as medians or averages. The chi-square test was applied to categorical data, and Student's *t-*test or Wilcoxon *t*-test were applied to continuous data. Odds ratios (ORs) with 95% confidence intervals (CIs) were assessed to determine the relationship between the changes in StO_2_, lactate and muscle injury markers. *P* < 0.05 was considered a statistically significant difference.

## Results

The characteristics of the 15 patients with complications of limb ischemia on the IABP side are shown in Table 1. Normalized single tissue oxygen saturation values, lactate, and myohemoglobin when ALI was diagnosed are shown in Table 2. There was no significant difference between the 2 groups.

**Table 1:** Demographics and clinical characteristics.

**Variables**	**No. (%) or median (interquartile range)**		***P*-value**
**Group**	**DDPC (6)**	**Conventional (9)**	
Age, years	48.2 (16–68)	54.1 (36–63)	0.25
Male	83.3%	77.8%	0.34
BMI^a^	27.2 (22.9–31.5)	24.7 (20.1–30.2)	0.41
Etiology			0.32
CAD	50%	55.6%	
VHD	50%	11.1%	
AD	0%	22.2%	
CHD	0%	11.1%	
**Complications**			
Hypertension	55.6%	33.3%	0.23
Diabetes mellitus	44.4%	33.3%	0.54
Smoking history	55.6%	66.7%	0.54
Femoral stenosis history	22.2%	16.7%	0.34
SOFA scores (when ALI was diagnosed)	12.9 (12–14)	12.7 (11–15)	0.55
Inotrope scores (when ALI was diagnosed)	61.7 (43–78)	54.4 (40–66)	0.36
ECMO Flow (LPM) (when ALI was diagnosed)	3.5 (3.2–3.8)	3.6 (3.5–3.9)	0.92

*BMI, body mass index; CAD, coronary artery disease; VHD, valvular heart disease; AD, aortic disease; CHD, congenital heart disease; SOFA, sequential organ failure assessment; Inotrope scores = dosage of dopamine (in μg/kg/min)+dosages of dobutamine (in μg/kg/min)+[dosages of epinephrine (in μg/kg/min+ norepinephrine (in μg/kg/min)] × 100+dosages of pituitrin (in u/min) × 100+dosages of milrinone (in μg/kg/min) × 15*.

**Table 2:** Clinical outcomes and ischemia indicator.

**Variables**	**No. (%) or mean** **±** **SD**		***P*-value**
**Group**	**DDPC (6)**	**Conventional (9)**	
ICU stay (days)	10.2 (8–13)	16.8 (3–45)	0.19
Acute compartment syndrome	0%	45.6%	0.03
Amputation	0%	11.1%	0.60
Weaning from ECMO	100%	33%	0.01
mortality	16.7%	88.9%	0.01
StO_2_ (IABP side when ALI was diagnosed)	31.5 (23–42)	35.0 (26–38)	1.00
StO_2_ (IABP side 6 h later)	61(55-66)^+^	35.0(28-39)	0.01
StO_2_ (IABP side 24 h later)	61.5 (52–63)^+^	35.0 (25–41)	0.01
Lactate (when ALI was diagnosed,mmol/L)	10.1 (5.7–17.1)	12.3 (5.4–16.6)	0.61
Lactate (6 h later,mmol/L)	9.0 (5.5–15.5)	13.0 (5.3–17)	0.61
Lactate (24 h later,mmol/L)	3.0 (2.5–7.8)^*^	12.6 (5.7–20)	0.07
Myohemoglobin (when ALI was diagnosed,ng/ml)	3,390.0 (3,390–3,390)	3,390.0 (3,280–3,390)	1.00
Myohemoglobin (6 h later,ng/ml)	3 390 (2,350–3,390)	3,390.0 (3,390–3,390)	1.00
Myohemoglobin (24 h later,ng/ml)	3,390 (1,880–3,390)	3,390.0 (3,106–3,390)	1.00
Myohemoglobin (48 h later,ng/ml)	475 (262–2,512)^#^	3,390 (578–3,390)	0.01

*ALI, acute limb ischemia; StO_2_, tissue saturation*.

* *compare with the Lactate (when ALI was diagnosed)*.

+ *compare with the StO_2_ (when ALI was diagnosed)*.

# *compare with the myohemoglobin (when ALI was diagnosed)*.

Overall, the in-hospital mortality for all patients was 60%, with a mean SOFA score of 12.8 ± 0.7. The mortality rate was significantly lower in the DDPC group (16.7, vs. 88.9%, *p* = 0.01). The ratio of successful ECMO weaning was significantly higher in the DDPC group than in the conventional group (100 vs. 33%, *p* = 0.01). Five patients in the conventional group developed osteofascial compartment syndrome and underwent incision and tensioning surgery (0 vs. 56% *p* = 0.03). One patient was amputated in the conventional group (0 vs. 11.1% *p* = 0.40). Furthermore, no recurrence of lower limb ischemia was noted in any patients in the DDPC group.

**Figure 2:**
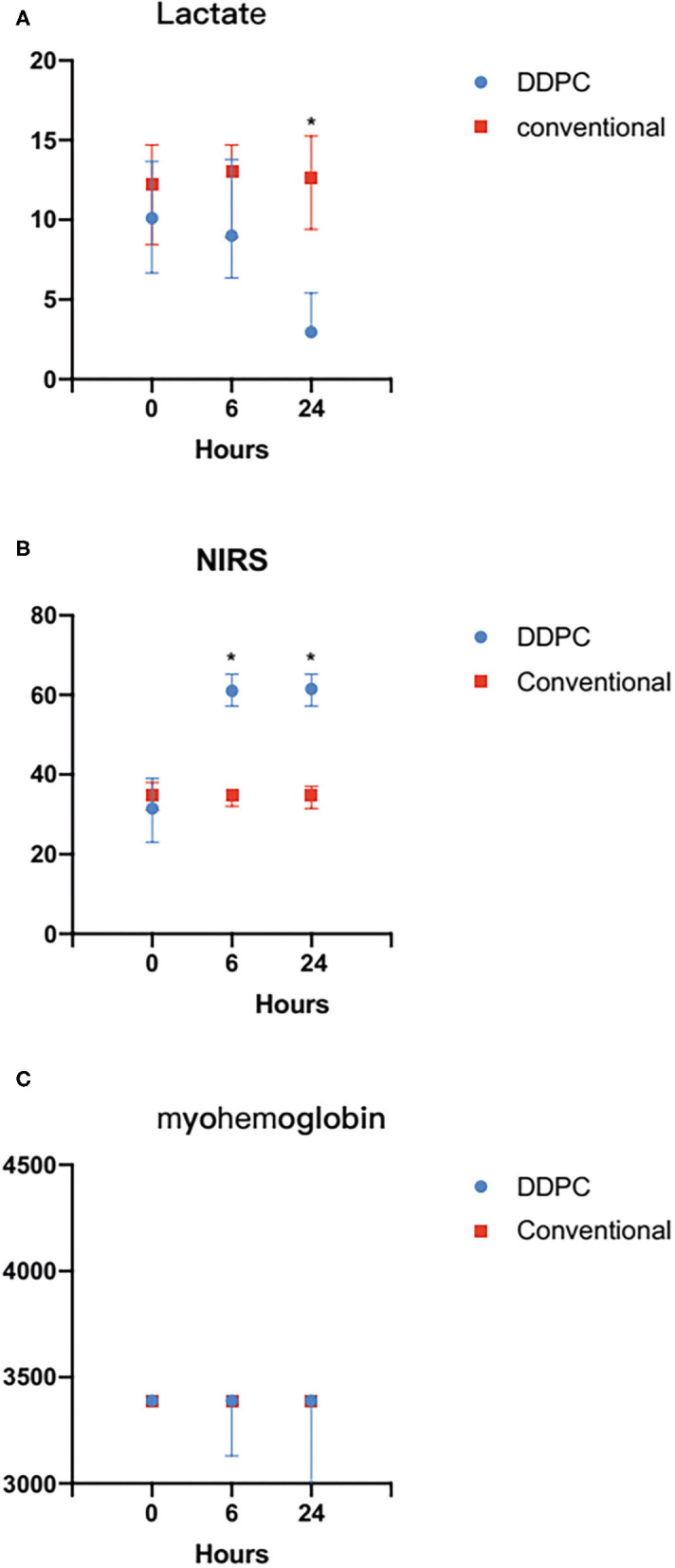
**(A)** Compared with the conventional treatment group, the StO2 was significantly increased 24 h after ALI in the DDPC group. **(B)** Compared with the conventional treatment group, the Lactate level decreased significantly 24 h after ALI in the DDPC group. **(C)** There was no significantly change in myohemoglobin within 24 h between 2 groups. *P < 0.05.

StO2 was noticeably increased 6 h later in the DDPC group. The lactate level was significantly increased 24 h later in the DDPC group, and the myohemoglobin was significantly reduced 48 h later in the DDPC group. These parameters did not change significantly in the conventional treatment group (Table 2; Figure 2).

## Discussion

In this study, we described a method to manage severe ALI developing on the IABP side in inpatients with ECMO and IABP support. To our knowledge, this is the first study comparing the outcomes of DDPC with conventional treatment in these patients. Our results showed that DDPC is associated with lower mortality. It could also reduce the rate of acute compartment syndrome.

The combined use of IABP in ECMO patients may reverse protracted aortic valve closure and impaired left ventricular unloading (11, 12). It can also increase the cerebral blood flow (13). However, Chen et al. reported that the concomitant use of IABP with ECMO did not appear to be associated with a dramatic change in survival outcomes. It increased the incidence of lower limb ischemia (14). ALI on IABP side patients has a poor prognosis with high mortality and a high incidence of acute compartment syndrome, despite the removal of IABP (15).

Haldun et al. used polytetrafluoroethylene external femoro-femoral bypass grafting in patients assisted with ECMO and IABP (16). However, this approach is complex and demonstrates a higher incidence of infection and thrombogenesis. In this study, we report our experience with DDPC in 6 patients with adult PCS shock receiving VA-ECMO with limb ischemia on the IABP side. This procedure is simple, with no bleeding or infection complications in the groin. None of the patients developed osteofascial compartment syndrome, and none needed amputation. The StO_2_ was notably higher 6 h after DDPC. The markers of muscle injury peaked within 48 h with lower limb blood supply improvement. There were no significant differences in SOFA scores or Inotrope scores between the two groups, indicating that the severity of the disease was similar between the two groups before grouping. There was a significant difference in prognosis between the two groups, and the mortality was significantly reduced in the DDPC group. The StO2 of the DDPC group was significantly increased after DDPC placement, and myoglobin and lactate were also significantly decreased. The results suggested that this might be due to the improvement of lower limb ischemia.

DDPC could be removed safely while ECMO was weaning. Contraction of the peripheral vessels caused by the large doses of vasoactive agents and poor cardiac output may be the main cause of limb ischemia in the early ECMO stage. With the stability of the circulation system and reduced use of vasoactive agents, the blood supply of the lower limbs gradually recovers.

Our study suggests that DDPC is a simple, safe, and effective method. It may play a vital role in patients with complications of lower limb ischemia on the IABP side following ECMO combined with IABP.

The study has several limitations. First, it was a non-randomized, retrospective, and observational study. The treatment strategies and monitoring methods that changed over time might have influenced the results of the study. Second, the number of patients included in this study was small, which may have prevented the detection of significant differences for other risk factors. Further multicenter studies are needed to corroborate the effectiveness of DDPC. Third, although pulse checks are still a routine diagnostic method for lower extremity ischemia in ECMO patients (17), advection perfusion of ECMO certainly has some influence on ABI, and whether this index needs to be corrected in ECMO patients needs further study to confirm.

## Conclusion

DDPC could be an effective method for lower limb ischemia on the IABP side in patients who received femoro-femoral VA-ECMO and IABP, and was associated with reduced mortality in these patients.

## Data Availability Statement

The original contributions presented in the study are included in the article/supplementary material, further inquiries can be directed to the corresponding author/s.

## Disclosure

All authors had freedom of investigation and full control of the design of the study, methods used, outcome parameters and results, analysis of data, and production of the written report.

## Author Contributions

MX is in charge of writing the articles. XH is in charge of providing idea. XT and LW are in charge of data analysis. HW, JW, and MJ are in charge of research design. DH is in charge of Chart production. All authors contributed to the article and approved the submitted version.

## Conflict of Interest

The authors declare that the research was conducted in the absence of any commercial or financial relationships that could be construed as a potential conflict of interest.

## Publisher's Note

All claims expressed in this article are solely those of the authors and do not necessarily represent those of their affiliated organizations, or those of the publisher, the editors and the reviewers. Any product that may be evaluated in this article, or claim that may be made by its manufacturer, is not guaranteed or endorsed by the publisher.

## Abbreviations

ABI: Ankle-brachial index
DDPC: Double distal perfusion catheter
EDPC: Extracorporeal membrane oxygenation side distal perfusion catheter
IDPC: Intra-aortic balloon pump side perfusion catheter
IABP: Intra-aortic balloon pumps
PCS: Postcardiotomy cardiogenic shock
SLI: Severe limb ischemia
ECMO: Extracorporeal membrane oxygenation
StO_2_: Tissue saturation
ICU: Intensive care unit
SOFA: Sequential organ failure assessment.
